# Current status and practical considerations of artificial intelligence use in screening and diagnosing retinal diseases: Vision Academy retinal expert consensus

**DOI:** 10.1097/ICU.0000000000000979

**Published:** 2023-07-13

**Authors:** Yu-Bai Chou, Aditya U. Kale, Paolo Lanzetta, Tariq Aslam, Jane Barratt, Carla Danese, Bora Eldem, Nicole Eter, Richard Gale, Jean-François Korobelnik, Igor Kozak, Xiaorong Li, Xiaoxin Li, Anat Loewenstein, Paisan Ruamviboonsuk, Taiji Sakamoto, Daniel S.W. Ting, Peter van Wijngaarden, Sebastian M. Waldstein, David Wong, Lihteh Wu, Miguel A. Zapata, Javier Zarranz-Ventura

**Affiliations:** a Department of Ophthalmology, Taipei Veterans General Hospital; b School of Medicine, National Yang Ming Chiao Tung University, Taipei, Taiwan; c Academic Unit of Ophthalmology, Institute of Inflammation & Ageing, College of Medical and Dental Sciences, University of Birmingham, Birmingham, UK; d Department of Medicine – Ophthalmology, University of Udine; e Istituto Europeo di Microchirurgia Oculare, Udine, Italy; f Division of Pharmacy and Optometry, Faculty of Biology, Medicine and Health, University of Manchester School of Health Sciences, Manchester, UK; g International Federation on Ageing, Toronto, Canada; h Department of Ophthalmology, AP-HP Hôpital Lariboisière, Université Paris Cité, Paris, France; i Department of Ophthalmology, Hacettepe University, Ankara, Turkey; j Department of Ophthalmology, University of Münster Medical Center, Münster, Germany; k Department of Ophthalmology, York Teaching Hospital NHS Foundation Trust, York, UK; l Service d’ophtalmologie, CHU Bordeaux; m University of Bordeaux, INSERM, BPH, UMR1219, F-33000 Bordeaux, France; n Moorfields Eye Hospital Centre, Abu Dhabi, UAE; o Tianjin Key Laboratory of Retinal Functions and Diseases, Tianjin Branch of National Clinical Research Center for Ocular Disease, Eye Institute and School of Optometry, Tianjin Medical University Eye Hospital, Tianjin; p Xiamen Eye Center, Xiamen University, Xiamen, China; q Division of Ophthalmology, Tel Aviv Sourasky Medical Center, Sackler Faculty of Medicine, Tel Aviv University, Tel Aviv, Israel; r Department of Ophthalmology, College of Medicine, Rangsit University, Rajavithi Hospital, Bangkok, Thailand; s Department of Ophthalmology, Kagoshima University, Kagoshima, Japan; t Singapore National Eye Center, Duke-NUS Medical School, Singapore; u Ophthalmology, Department of Surgery, University of Melbourne, Melbourne, Australia; v Centre for Eye Research Australia, Royal Victorian Eye and Ear Hospital, East Melbourne, Victoria, Australia; w Department of Ophthalmology, Landesklinikum Mistelbach-Gänserndorf, Mistelbach, Austria; x Unity Health Toronto – St. Michael's Hospital, University of Toronto, Toronto, Canada; y Macula, Vitreous and Retina Associates of Costa Rica, San José, Costa Rica; z Ophthalmology Department, Hospital Vall d’Hebron; aa Hospital Clínic de Barcelona, University of Barcelona, Barcelona, Spain

**Keywords:** artificial intelligence, diagnosis, retina, retinal imaging

## Abstract

The application of artificial intelligence (AI) technologies in screening and diagnosing retinal diseases may play an important role in telemedicine and has potential to shape modern healthcare ecosystems, including within ophthalmology.

**Recent findings:**

In this article, we examine the latest publications relevant to AI in retinal disease and discuss the currently available algorithms. We summarize four key requirements underlining the successful application of AI algorithms in real-world practice: processing massive data; practicability of an AI model in ophthalmology; policy compliance and the regulatory environment; and balancing profit and cost when developing and maintaining AI models.

**Summary:**

The Vision Academy recognizes the advantages and disadvantages of AI-based technologies and gives insightful recommendations for future directions.

## INTRODUCTION

The initial concept of artificial intelligence (AI) was first coined as far back as 1956 [1]. The concept of “machine learning” and “deep learning” (DL) were proposed subsequently and demonstrated great potential in computer learning and decision-making via various data training techniques [2–4]. AlexNet, which won the ImageNet Large Scale Visual Recognition Challenge in 2012, set a milestone for DL algorithms to handle large imaging data sets [5]. Since then, the application of DL algorithms to color fundus photography has been adopted for the diagnosis and monitoring of many retinal diseases, including diabetic retinopathy (DR) [6–10], age-related macular degeneration (AMD) [10,11], and retinopathy of prematurity [12]. Moreover, wide-field fundus photography and autofluorescence imaging have been used to differentiate not only referable DR and AMD but retinal vein occlusion, pathologic myopia, retinal detachment, vitreomacular interface disease, pathologic myopia, sickle cell retinopathy, and inherited retinal diseases by DL-based models [13–19].

In recent years, many studies have applied AI algorithms to the most modern retinal imaging modalities, including optical coherence tomography, to detect or quantify an array of retinal features of interest (e.g. retinal fluid) [20–23]. AI-enabled technologies, which use not only different types of images but also different data modalities (e.g. structured medical data [24] and genomics data sets [25,26]), have also proven to demonstrate robust outcomes.

Such AI-enabled technologies have potential to be implemented into clinical practice in several ways. The use of AI technologies to screen or classify retinal diseases may play a role in telemedicine. They may also assist healthcare providers with greater speed, repeatability, reproducibility, and consistency than human graders. Uniting clinicians with AI systems has been proven to be synergistic, achieving better performance than either alone [27]. Therefore, AI-enabled technology can help clinicians achieve rapid and accurate decision-making.

Academic institutions and technology companies (e.g. Google) increasingly engage in AI research and boost their investment and involvement in this field [7]. Furthermore, the U.S. National Science and Technology Council's Committee on Technology noted that investments from the U.S. government in AI-enabled technologies were nearly $1.1 billion in 2015 and continue to increase [28].

The key issues for deploying AI technologies in telemedicine or healthcare systems may have a profound and lasting influence on near-future practice in ophthalmology.

In this article, we summarize four key requirements surrounding the application and execution of AI-enabled technology for diagnosis and screening in retinal diseases in real-world practice. Informing and operationalizing an AI healthcare system includes processing large data sets, practicability in ophthalmology, policy compliance and regulatory environment, and balancing profit and cost in adopting AI-enabled technologies. 

**Box 1:**
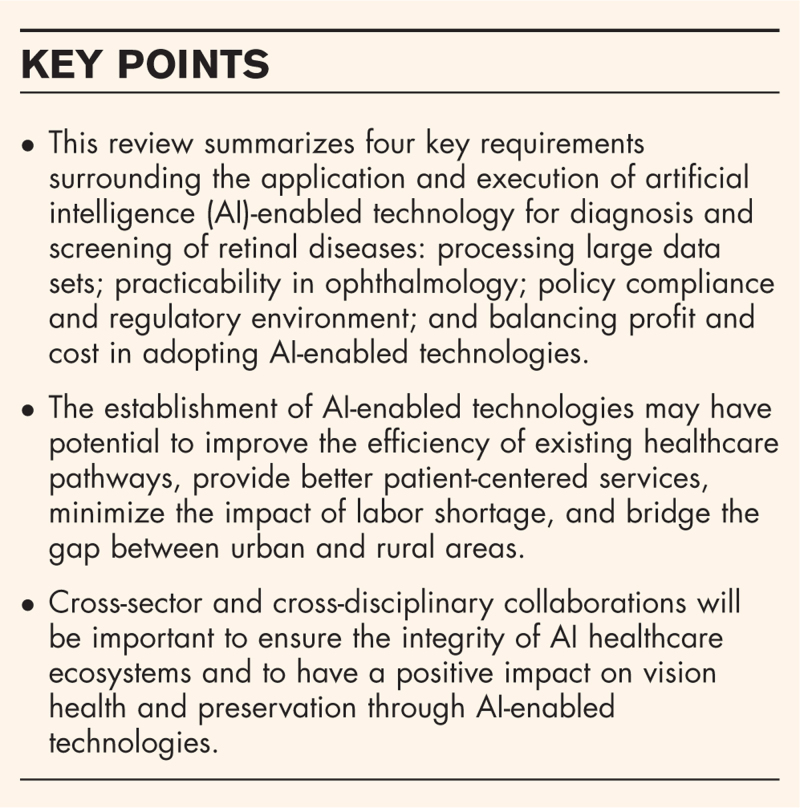
no caption available

## PROCESSING LARGE DATA SETS: DATA STANDARDIZATION, SHARING, AND SAFETY

Data processing is crucial before developing an AI model. It includes data standardization, data sharing system, and the maintenance of data privacy in the infrastructure of AI systems.

### Data standardization in ophthalmology

The high dependency of modern ophthalmology on imaging makes it an attractive field for the development of AI models. However, the diversity of proprietary devices, image acquisition, and data storage processes poses a barrier to research teams. The need for data standardization has become pivotal, not only for expanding the scale of AI models but for providing more effective ways to achieve clinical benefits. In 2021, the American Academy of Ophthalmology suggested that manufacturers of ophthalmic devices should standardize the format of digital images, integration of medical data, and picture archiving to comply with the 12 Digital Imaging and Communications in Medicine standards, developed by the American Academy of Ophthalmology in collaboration with manufacturers [29]. Such standardization of file formats in proprietary databases may streamline further analysis and increase the interoperability of different cameras or devices [30].

### Data sharing and privacy: are the protections adequate?

As of 2021, nearly 94 ophthalmic data sets containing more than 500 000 images were openly accessible [31]. The transparency, accessibility, and limitations of each data set should be carefully shared and reported because these factors can affect the ground truth of image processing, not to mention the extensibility of AI algorithms.

The shared data should be deidentified and anonymized to comply with privacy and cybersecurity frameworks. Data privacy poses challenges in technological, legal, and ethical fields. It can be difficult to precisely define data privacy because traditional deidentification is vulnerable to linkage attacks from intended third parties [32]. Moreover, fundus images are now considered uniquely recognizable information due to AI-enabled technology [33]. Awareness of such biometric identification, which may breach privacy rights, is crucial in data processing. Synthetic data generation (e.g. generative adversarial network) is a feasible way of creating plausible images for AI training while also maintaining confidentiality and provides an anonymization technique for data privacy [34,35].

Data privacy may also be addressed by collating all relevant data into trusted research environments or data decentralization. In federated analysis, the algorithmic code is sent to each data site for individual analysis; then the results are brought back to the central site for aggregation and further analysis [36]. Recently, swarm learning was proposed to provide blockchain-based peer-to-peer data security. In contrast to federated learning, which requires a central analytic server, swarm learning produces complete data decentralization [37]. Compared with traditional centralized data, these new AI-enabled technologies with data decentralization can preserve privacy by retaining the data in each institution while still achieving similar outcomes [38].

### Ethical considerations and legal liability

In 2021, the American Academy of Ophthalmology Committee on Artificial Intelligence raised three ethical concerns: transparency, meaning the adequate explanation or interpretation of the AI model; responsibility, which addresses moral or legal concerns; and scalability of implementation of AI models, which depends on equality of data distribution and potential systemic bias in AI models [39^▪^]. AI technologies may change relationships between physicians, healthcare organizations, and patients. However, there is still no universal guidance for legal liability. The American Medical Association recommends that developers should take legal liability and maintain insurance for systemic failure or misdiagnosis from an autonomous AI system [40]. The more autonomous the design of an AI model is, the more reinforcement is needed in terms of legal liability [41]. Different entities may share liability: physician errors belong to negligence liability; healthcare organization errors belong to vicarious liability; and manufacturer errors are attributed to product liability and incomplete disclosure of the actual functions and limitations of an AI model [42]. These entities should take responsibility to compensate for financial and physical loss to the injured party [43].

## PRACTICABILITY OF ARTIFICIAL INTELLIGENCE IN CLINICAL TRIALS AND TELEMEDICINE

As the field of ophthalmic AI evolves, the quality of reporting results from different AI systems may be discrepant and incomprehensive. It is necessary to have consensus in determining adequate description, translation, and appraisal of ophthalmic AI research to ensure robust algorithms and generalizability into real-world settings.

### Role of physicians and researchers in clinical trials

In 2020, CONSORT-AI (CONsolidated Standards Of Reporting Trials–Artificial Intelligence) and SPIRIT-AI (Standard Protocol Items: Recommendations for Interventional Trials–Artificial Intelligence) were announced to provide reporting guidance in AI-related clinical trials [44^▪▪^]. Subsequently, other reporting guidelines (i.e. STARD-AI, DECIDE-AI, and TRIPOD-AI) further emphasized the transparency of AI technologies in healthcare. These have presented new standards for evaluating the results from AI-related clinical trials [44^▪▪^,^45^^▪▪^,^46^] and allowed for clear evidence generation and decision making.

### Role of healthcare providers in telemedicine

Healthcare equity has been a major concern due to the imbalanced distribution of resources between urban and rural areas. The deployment of AI screening algorithms accompanied by well-established infrastructure of cybersecurity, including cloud-based systems or even home-based devices, can facilitate the adoption of AI technologies and reduce the medical resources gap between urban and rural areas. On the contrary, the quality of data-driven technologies may be affected by the inequality of data distribution (e.g. differences between racial and ethnic groups), which reduces the generalizability of the AI models to specific populations due to the scarcity of related data. Overemphasizing an AI system without carefully considering the condition of health data poverty has potential to cause harm [47]. Even if AI-enabled technologies represent an opportunity to overcome some challenges in rural areas, the general application and clinical interpretation of AI models should be treated with caution.

## POLICY COMPLIANCE AND THE REGULATORY ENVIRONMENT IN ARTIFICIAL INTELLIGENCE

Regulatory considerations for AI medical devices or software should include data security and sourcing, the design and development of algorithms, and evidence generation from AI-enabled technologies. Governmental regulatory bodies should provide clear guidance regarding the evidence requirements for AI medical devices and, to streamline the process of training, continuous education, and relicensing [43].

For data privacy and confidentiality, in 2016 and 2017 the EU introduced the General Data Protection Regulation 2016/679 [48], the EU Network and Information Security Directive 2016/1148 [49], and the Medical Devices Regulation 2017/745 [50,51]. In the United States, the Health Insurance Portability and Accountability Act of 1996 covers confidentiality issues in medical data [43].

For adopting and marketing AI-based instruments and algorithms, different regulatory groups are responsible for ensuring the security and safety of the products. The U.S. Food and Drug Administration (FDA) announced the Digital Health Innovation Action Plan to streamline the premarket review and to outline its approach to AI-based frameworks, known as “software as medical device” [52], which is a term defined by the International Medical Device Regulators Forum [42,53]. With regards to the risk of AI products, three risk classes represent safety and effectiveness for patients: class I (low risk); class II (moderate risk); and class III (high risk). AI medical devices must be evaluated rigorously through appropriate regulatory pathways. FDA regulatory pathways include the 510(k), De Novo Classification, and Premarket Approval. The appropriate pathway is determined by the risk class of the AI medical device and whether there is a predicate device available on the market already [54].

In contrast to the United States, which adopts more market-oriented regulations, the EU takes a more customer-oriented approach to build the framework for AI-enabled technologies, with the Conformitè Européenne playing a critical role in the licensing of AI products [54]. Many other regulatory parties from other countries are involved in licensing AI-enabled technologies, such as the UK Conformity Assessed mark and the Chinese National Medical Products Administration.

## BALANCING PROFIT AND COST IN ADOPTING ARTIFICIAL INTELLIGENCE-ENABLED TECHNOLOGIES FOR REAL-WORLD IMPLEMENTATION

For adoption of AI technologies, achieving a practical balance between profit and cost is another important issue. Premarketing costs include significant effort and workforce in data collection, research development, and validation. Postmarketing costs include upgrading software, sustaining hardware over the long term, training operators, and incorporating new patient data [55,56]. Funding support can ensure the adoption and ongoing maintenance of AI services, but only when a balance between profit and cost is reached can the development of AI steadily progress.

The cost of adopting AI-enabled technologies should be balanced between manufacturing price and reimbursements as it is considered by and for healthcare providers. In the example of Singapore's national DR screening programs, cost savings of approximately U.S. $21.9 million were achieved for a group of 170 000 patients with diabetes who underwent AI-assisted screening [57]. In the UK, the National Institute for Health and Care Excellence set up the Evidence Standards Framework to enable assessment of digital health technologies and guide government, developers, and healthcare providers on the level of evidence for economic and clinical evaluation [58]. Such guidance can motivate the application and prevent the overuse of AI-enabled technologies in the healthcare ecosystem. Government, industry, and academia will be the iron triangle for the future implementation of AI-enabled technologies in screening and diagnosing retinal diseases.

## CURRENT DEVELOPMENTS, FUTURE DIRECTIONS, AND VISION ACADEMY RECOMMENDATIONS

Current AI-enabled systems with regulatory compliance are outlined in Table 1, with further details on their performance provided in Table 2.

**Table 1:** Summary of current AI systems with regulatory approval for different retinal diseases

AI system	Type of application	Year approved	Target disease	Imaging modality	Target user	Regulatory status in the United States, Europe, and elsewhere
Intelligent Retinal Imaging Systems (IRIS; Intelligent Retinal Imaging Systems, Pensacola, FL, USA) [59]	Cloud-based algorithm	2015	Screen for DR	Fundus photography	Healthcare provider	FDA clearance (class II)
Automated Retinal Disease Assessment (ARDA; Google LLC, Mountain View, CA, USA) [60]	Cloud-based algorithm	2016	Screen for referable DR	Fundus photography	Healthcare provider	CE mark
SELENA+ (EyRIS Pte Ltd, Singapore) [61]	Cloud-based algorithm	2019 and 2020	Screen for vision-threatening DR, suspected glaucoma, AMD	Fundus photography	Healthcare provider	CE mark, HAS approval (Singapore)
IDx-DR (Digital Diagnostics Inc., Coralville, IA, USA) [62]	Device and cloud-based algorithm	2018	Screen for referable DR, including DME	Fundus photography	Healthcare provider	FDA approval
Medios AI (Remidio Innovative Solutions Pvt Ltd., Karnataka, India) [63]	Offline AI algorithm	2023	Screening for referable DR and referable glaucoma	Fundus photography from smartphone-based camera	Healthcare provider	CE mark
RetCAD (Thirona Retina BV, Nijmegen, Netherlands) [64]	Cloud-based algorithm	2022	Screen for referable DR, AMD	Fundus photography	Healthcare provider	CE mark
EyeArt (Eyenuk, Inc., Woodland Hills, CA, USA) [65]	Cloud-based algorithm	2015 and 2020	Screen for referable DR	Fundus photography	Healthcare provider	FDA clearance (class II), CE mark
VUNO Med-Fundus AI (VUNO Inc., Seoul, Korea) [66]	Cloud-based algorithm	2020	DR, AMD, glaucoma	Fundus photography	Healthcare provider	CE mark (class IIa), MFDS approval (Korea, class III), HAS approval (Singapore)
THEIA (Toku Eyes, Auckland, New Zealand) [67]	Cloud-based algorithm	2020	Smoking status, AMD, DR, cataract	Fundus photography, OCT-A	Healthcare provider	In progress
iPredict (iHealthScreen Inc., Richmond Hill, NY, USA) [68]	Cloud-based algorithm	2021 and 2022	DR, AMD, glaucoma	Fundus photography, OCT	Healthcare provider	CE mark, TGA approval (Australia)
Notal Home OCT (Notal Vision, Inc., Manassas, VA, USA) [69]	In-home device and cloud-based algorithm	2018	Neovascular AMD	OCT	Patient-driven healthcare	FDA Breakthrough Device Designation
OphtAI (Evolucare/ADCIS, Villers-Bretonneux, France) [70]	Cloud-based algorithm	2019	DR, DME, AMD, glaucoma	Fundus photography	Healthcare provider	CE mark, HC approval (Canada), FDA in progress
Retmarker (Retmarker, SA, Taveiro, Portugal) [71]	Cloud-based algorithm	2010	DR, AMD	Fundus photography	Healthcare provider	CE mark (class IIa), TGA approval (Australia)
RetinaLyze (RetinaLyze System A/S, Hellerup, Denmark) [72]	Cloud-based algorithm	2021	DR, dry AMD, glaucoma	Fundus photography, OCT	Healthcare provider	CE mark (class I, self-certified)
RetinAI Discovery (RetinAI Medical AG, Bern, Switzerland) [73]	Cloud-based algorithm	2022	AMD, DR, DME, RVO	Fundus photography, OCT	Healthcare provider	FDA clearance (class II), CE mark

*Note:* All medical devices approved by the FDA or accredited by the CE mark from January 2015 to November 2022 were collected. These devices were searched for in the European Database on Medical Devices (EUDAMED) database [74], the FDA website (on the webpage of Artificial Intelligence and Machine Learning-Enabled Medical Devices) [75], and the FDA 510(k) Premarket Notification [76]. ARDA, Automatic Retinal Disease Assessment; DME, diabetic macular edema; HAS, Health Sciences Authority (Singapore); HC, Health Canada; IRIS, Intelligent Retinal Imaging Systems; MFDS, Ministry of Food and Drug Safety (Korea); OCT, optical coherence tomography; OCT-A, optical coherence tomography angiography; RVO, retinal vein occlusion; SELENA, Singapore Eye LEsioN Analyzer; TGA, Therapeutic Goods Administration.

**Table 2:** Description and notification of current AI systems

AI system	Description and notification
IRIS (Intelligent Retinal Imaging Systems, Pensacola, FL, USA)	IRIS is an FDA class II cleared medical system that has a moderate risk to consumers and must demonstrate that it is “substantially equivalent” to similar products. The IRIS program is a cloud-based platform to screen for vision-threatening DR, with sensitivity and specificity of 66.4% and 72.8%, respectively [77]
ARDA (Google LLC, Mountain View, CA, USA)	ARDA is a DL algorithm developed by Google Health from >128 000 retinal photographs of patients from the United States and India and validated in >10 000 photographs from the UK to detect referrable and sight-threatening DR [7]. The validation study of ARDA was the first showing robust performance of DL to detect referrable DR with >95% of both sensitivity and specificity [7]. Later, ARDA was prospectively validated in India [78] and a nationwide screening program in Thailand [79]
SELENA+ (EyRIS Pte Ltd, Singapore)	The Singapore Eye Research Institute and Singapore National Eye Center has developed a DL-based algorithm, SELENA+, to screen for referable DR, vision-threatening DR, DR-related vascular risk factors, suspected glaucoma, and late-stage AMD. It is a multicenter collaborative research effort with half a million retinal images from people of different ethnicities such as Caucasians from Australia and the United States, and Singapore Chinese, Malayans, Indians, Chinese, individuals from Hong Kong, Mexicans, Hispanics, and African Americans. Real-world application and clinical translation of SELENA+ has been integrated into the Singapore Integrated Diabetic Retinopathy Programme in recent years. SELENA+ has significant diagnostic performance in DR, with sensitivity of 91%, specificity of 90%, and area under the curve of 0.93 [80]
IDx-DR (Digital Diagnostics Inc., Coralville, IA, USA)	IDx-DR was the first FDA-approved ophthalmic device to autonomously detect DR, including DME. It can analyze retinal images, detect vision-threatening DR, and provide referral recommendations [6,81]. The external validation to detect referable DR showed sensitivity and specificity of 91% and 84%, respectively [79]
Medios AI (Remidio Innovative Solutions Pvt Ltd., Karnataka, India)	Medios AI is an integrated offline system with a Remidio smartphone-based, nonmydriatic retinal camera to detect referable DR. The fundus images can be captured by minimally trained healthcare providers. The sensitivity and specificity of diagnosing referable DR were 100% and 88.4%, respectively [82]
RetCAD (Thirona Retina BV, Nijmegen, Netherlands)	This commercially available DL algorithm can determine referable DR and AMD based on a dataset of CFPs to reduce the workload of screening programs by up to 96%, with sensitivity of 90.53% and specificity of 97.13%. Patients’ CFPs can be captured by camera and then transferred to the Thirona server for analysis. The examination report will provide referable suggestions and visualization of heatmaps [83]
EyeArt (Eyenuk, Inc., Woodland Hills, CA, USA)	This cloud-based autonomous AI system can detect more-than-mild DR and vision-threatening DR by submitting fundus photography to the platform. It is designed to work with various types of retinal cameras. It assesses the quality of uploaded images and explains the reasons behind grading. This algorithm can provide the grading of DR and report the results for each eye based on the UK National Health Service diabetic eye screening program scale. The sensitivity and specificity showed 96% and 98%, respectively [84]
VUNO Med-Fundus AI (VUNO Inc., Seoul, Korea)	The AI-based VUNO Med-Fundus AI analyzes CFP to detect multiple retinal lesions (areas under receiver operating characteristic curves for all findings were at 96.2%) [85]. The area under the receiver operating characteristic curves for DR-related findings was 95%. It was approved as a class III medical device by the Ministry of Food and Drug Safety in Korea
THEIA (Toku Eyes, Auckland, New Zealand)	The New Zealand company Toku Eyes developed THEIA, an AI platform for cloud-based multimodal image analysis of referable DR and AMD. The THEIA system was developed from two of the largest screening data sets in Auckland, New Zealand: the Auckland District Health Board and the Counties Manukau District Health Board. It can analyze color fundus images, OCT, and OCT-A to provide results about referable DR (sensitivity of 93% and specificity of 63%) and intermediate dry AMD (accuracy of 96%) [86,87]. This AI system is considered to be useful in reducing the workload in the New Zealand National Diabetic Retinopathy Screening Program [88]
iPredict (iHealthScreen Inc., Richmond Hill, NY, USA)	The iPredict AI Eye Screening System offers fully automated diagnosis of referable DR (sensitivity of 97.0% and specificity of 96.3%) and AMD (sensitivity of 86.6% and specificity of 92.1%) by analyzing CFPs [89]
Notal Home OCT (Notal Vision, Inc., Manassas, VA, USA)	Notal Home OCT, the first FDA-cleared in-home OCT device, which includes an AI algorithm and monitoring center, is designed to detect AMD. The imaging quality showed great correlation with in-office OCT for detecting the presence of fluid in 95% agreement with human graders [90]. A patient's ability to use an in-home setting for self-imaging without training demonstrated good capacity with a 95% success rate [91]
OphtAI (Evolucare/ADCIS, Villers-Bretonneux, France)	OphtAI DR is a semiautomatic AI algorithm that assesses the pathologic lesions and grading of DR and detects AMD and glaucoma. In a multicenter, head-to-head, real-world validation study to compare different algorithms in detecting DR, the OphtAI DR algorithm provided better results (sensitivity of 80.47% and specificity of 81.28%) than an ophthalmologist [92]. It is also deemed clinically safe and economically efficient in reducing the costs by more than U.S. $15 per patient [92]
Retmarker (Retmarker, SA, Taveiro, Portugal)	This AI technology can provide screening for DR and AMD by annotating pathologic lesions, such as microaneurysms, drusen, hypopigmentation, hyperpigmentation, and geographic atrophy [93]. The sensitivity in classifying DR is 73.0% for any DR, 85.0% for referable DR, and 97.9% for proliferative DR [93]. The screening performance of Retmarker appeared to vary with patients’ age, ethnicity, and camera type. In economic analysis, the Retmarker was more cost effective than manual grading [93]
RetinaLyze (RetinaLyze System A/S, Hellerup, Denmark)	The RetinaLyze system is a screening software that can detect DR on nonmydriatic CFPs (sensitivity of 89.9% and specificity of 85.7%) [94]. It can detect DR lesions, including microaneurysms and minor hemorrhages (specificity of 71.4%) [95]. It can also evaluate biological aging [96] and hemoglobin on optic disc photographs [97]
RetinAI Discovery (RetinAI Medical AG, Bern, Switzerland)	The Discovery platform can analyze medical data and ophthalmic images such as OCT scans and CFP from a variety of devices. It can help automatically detect the location of the fovea (mean total location error of 0.101 mm), the quantification of pathologic fluid, and the segmentation of atrophic retina on OCT in patients with geographic atrophy [98–100]. It can detect and quantify fluid from DR, DME, AMD, and RVO. The performance of the AI system showed that the accuracy, specificity, and sensitivity for intraretinal fluid was 0.87, 0.88, 0.84 and 0.93, 0.95, 0.93 and for subretinal fluid was 0.93, 0.93, 0.93 and 0.95, 0.95, 0.95 in the AMD and DME cohorts, respectively [101^▪^,^102^]

*Note:* All medical devices approved by the FDA or accredited by the CE mark from January 2015 to January 2023 were collected. These devices were searched for in the European Database on Medical Devices (EUDAMED) database [74], the FDA website (on the webpage of Artificial Intelligence and Machine Learning-Enabled Medical Devices) [75], and the FDA 510(k) Premarket Notification [76]. The approved devices were summarized and their performance in related trials was searched for in PubMed, with the data source cited as the reference. ARDA, Automatic Retinal Disease Assessment; CFP, color fundus photograph; DME, diabetic macular edema; IRIS, Intelligent Retinal Imaging Systems; OCT, optical coherence tomography; OCT-A, optical coherence tomography angiography; RVO, retinal vein occlusion; SELENA, Singapore Eye LEsioN Analyzer.

The Vision Academy recognizes the advantages of AI technology and recommends the use of them to be of additive and synergistic value to current standards of care. In terms of applying such technologies in diagnosing and screening retinal diseases, we summarize the following directions and emphasize several viewpoints important for the future.

### Recommendation 1: integration of meta-data and data sets

The integration of meta-data, including multimodal images and structured clinical information from multiple data sets with different ethnic groups, and establishment of a data processing and sharing system will empower data-driven AI technologies in ophthalmic practice. Ongoing research will be needed to build up data storage and sharing systems in a cybersecurity framework for broader use.

### Recommendation 2: data privacy versus transparency – a balance or conflict?

While retinal images possess biometric information that could be reidentified by AI technologies, care should be taken when collecting and processing these images. Some novel learning tasks (e.g. generative adversarial networks) can obscure bioidentical information or even provide unsupervised models for small-scale data sets. The question of how to universalize data formats will be one of the key factors for extending the scalability and generalizability of AI-enabled technologies.

The complexity and inexplicability of AI are encompassed in the term “black box phenomenon.” Black box algorithms have potential to cause misuse of AI in healthcare ecosystems [74,101^▪^]. Transparency of the algorithms is therefore another critical point to overcome users’ hesitation. Maintaining adequate balance between data privacy and transparency should be a concern in the application of AI-enabled technologies.

### Recommendation 3: implementation of artificial intelligence in clinical practice – replacement or rectification?

The role of AI-enabled technologies in the real world is not to replace ophthalmologists but to assist them and to hybridize both AI models and human experience for making more efficient and accurate decisions. Such time-saving abilities could streamline medical procedures and give clinicians more time to communicate with their patients. Improper implementation of AI could be harmful to doctor–patient relationships and could affect patients’ trust if AI algorithms were used only for improving workflow but not patient care.

### Recommendation 4: ethical concerns and regulatory issues

A key hurdle in deploying AI-enabled technologies in clinical practice is the fear of making an incorrect decision and harming patients. Legal liability should be well defined as the implementation of AI becomes more popular. Such liability should only be at the precise claim of screening targeted diseases. Unlike retinal specialists, the developers of an AI model should only be liable for the designed algorithm for screening specific diseases. Healthcare providers should still take full responsibility for being aware of the capacity of AI models.

The legal boundaries between developers and healthcare providers are still unresolved, and legislative and governance systems need to be more established to refine liability rules and the regulatory environment. Policy and specific authorities should be set up not only for verification of AI models but for data security and legal liability. Cross-sector and cross-disciplinary collaborations will be important to ensure the integrity of AI healthcare ecosystems.

### Recommendation 5: long-term basis

Continuing education, promotion of practical application, and user-friendly, understandable interfaces for healthcare providers are equally important to streamline the workflow and broaden the applicability of AI systems. Communication and collaboration between cross-functional teams, including ophthalmologists, optometrists, computer scientists, statisticians, data scientists, patient organizations, and engineers, can have a positive impact on vision health and preservation through AI-enabled technologies.

## CONCLUSION

The establishment of AI-enabled technologies may have potential to improve the efficiency of existing healthcare pathways, provide better patient-centered services, minimize the impact of labor shortage, and bridge the gap between urban and rural areas. However, no advancement in clinical practice is flawless, so it is necessary for healthcare providers and legislators to be aware of the limitations of AI-enabled devices.

## Acknowledgements

*Editorial assistance was provided by Elle Lindsay, PhD, Macha Aldighieri, PhD, and Rachel Fairbanks, BA (Hons), of Complete HealthVizion, Ltd, an IPG Health Company, funded by Bayer Consumer Care AG, Pharmaceuticals Division, Basel, Switzerland.*

### Financial support and sponsorship

*The Vision Academy is a group of over 100 international ophthalmology experts who provide guidance for best clinical practice through their collective expertise in areas of controversy or with insufficient conclusive evidence. The Vision Academy is funded and facilitated by Bayer. The opinions and guidance of the Vision Academy outputs are those of its members and do not necessarily reflect the opinions of Bayer.*

*Financial arrangements of the authors with companies whose products may be related to the present report are listed in the “Conflicts of interest” section, as declared by the authors.*

### Conflicts of interest

*Yu-Bai Chou is a consultant for Alcon and Bayer. Paolo Lanzetta is a consultant for Aerie, AbbVie, Apellis, Bausch & Lomb, Bayer, Biogen, Boehringer Ingelheim, Genentech, Novartis, Ocular Therapeutix, Outlook Therapeutics, and Roche. Tariq Aslam is a consultant for, and has received grants, speaker fees, and honoraria from, Allergan, Bayer Pharmaceuticals, Canon, NIHR, Roche, and Topcon. He is also a board member for the Vision Academy, Macular Society, and Fight for Sight charity. Jane Barratt has received honoraria from Bayer. Carla Danese is a consultant for Bayer. Bora Eldem is a consultant for Allergan, Bayer, Novartis, and Roche. Nicole Eter is an advisor for AbbVie, Alcon, Apellis, Bayer, Biogen, Janssen, Novartis, and Roche and has received speaker fees from AbbVie, Apellis, Bayer, Novartis, and Roche and research grants from Bayer and Novartis. Richard Gale is a consultant for AbbVie, Allergan, Apellis, Bayer, Biogen, Boehringer Ingelheim, Notal, Novartis, Roche, and Santen and has received research grants from Bayer, Novartis, and Roche. Jean-François Korobelnik is a consultant for Allergan/AbbVie, Apellis, Bayer, Carl Zeiss Meditec, Janssen, Nano Retina, Roche, and Théa and is a member of the Data and Safety Monitoring Boards for Alexion and Novo Nordisk. Igor Kozak is a consultant for Alcon, Bayer, and Novartis. Anat Loewenstein is a consultant for Allergan, Annexon, Bayer Healthcare, Beyeonics, Biogen, ForSight Labs, IQVIA, Iveric Bio, Johnson & Johnson, MJH Events, Nano Retina, Notal Vision, Novartis, Ocuphire Pharma, OcuTerra, OphtiMedRx, Roche, Ripple Therapeutics, Syneos, WebMD, and Xbrane. Paisan Ruamviboonsuk is a consultant for, and has received research funds from, Bayer and Roche. Taiji Sakamoto is a consultant for Bayer Yakuhin, Boehringer Ingelheim, Chugai, Nidek, Nikon, Novartis, Santen, and Senju. Daniel S.W. Ting has received research grants from the National Medical Research Council Singapore, Duke-NUS Medical School Singapore, and Agency for Science, Technology and Research Singapore. Peter van Wijngaarden is the co-founder of Enlighten Imaging, an early-stage medical technology start-up company devoted to hyperspectral retinal imaging and image analysis, including the development of AI systems, and has received research grant support from Bayer and Roche and honoraria from Bayer, Mylan, Novartis, and Roche. Sebastian M. Waldstein is a consultant for Apellis, Bayer, Boehringer Ingelheim, Novartis, Roche, and Santen. David Wong is a consultant for AbbVie, Alcon, Apellis, Bayer, Bausch Health, Biogen, Boehringer Ingelheim, Novartis, Ripple Therapeutics, Roche, Topcon, and Zeiss, has received financial support (to institution) from Bayer, Novartis, and Roche, and is an equity owner at ArcticDx. Lihteh Wu is a consultant for Bayer, Lumibird Medical, Novartis, and Roche. Miguel A. Zapata is a consultant for Novartis and Roche, has received grants and speaker fees from DORC, Novartis, and Roche, honoraria from Alcon, Bayer, DORC, Novartis, and Roche, has served on advisory boards for Novartis and Roche, has received equipment from Allergan, and has stock or stock options in UpRetina. Javier Zarranz-Ventura has received grants from AbbVie, Allergan, Bayer, Novartis, and Roche, has served on scientific advisory boards for AbbVie, Allergan, Bayer, Novartis, and Roche, and has been a speaker for AbbVie, Alcon, Alimera Sciences, Allergan, Bausch & Lomb, Bayer, Brill Pharma, DORC, Esteve, Novartis, Roche, Topcon Healthcare, and Zeiss. Aditya U. Kale, Xiaorong Li, and Xiaoxin Li have no conflicts of interest to report.*

## REFERENCES AND RECOMMENDED READING

Papers of particular interest, published within the annual period of review, have been highlighted as:

▪ of special interest
▪▪ of outstanding interest
